# Factors associated with health facility delivery among reproductive age women in Nepal: an analysis of Nepal multiple indicator cluster survey 2019

**DOI:** 10.1186/s12913-022-08822-5

**Published:** 2022-11-28

**Authors:** Naba Raj Thapa, Shanti Prasad Upreti

**Affiliations:** Department of Population Studies, Ratna Rajyalaxmi Campus, Tribhuvan University, Kathmandu, Nepal

**Keywords:** Antenatal care, Home delivery, Health facility delivery, Maternal mortality, Multivariate analysis

## Abstract

**Background:**

Despite existing efforts to improve maternal health in Nepal, delivery in a health facility with skilled providers is still a major health concern in Nepal. This study aimed to examine the factors associated with delivery in a health facility with skilled providers among women aged 15–49 years in Nepal.

**Methods:**

This study used data from Nepal Multiple Indicator Cluster Survey 2019, a nationally representative cross-sectional survey. This survey was a two-stage, stratified cluster sampling. A total of 1,950 married women aged 15–49 years who had at least one live birth in the last two years preceding the survey were included in the analysis. Bivariate and multivariate logistic regression analyses were performed in this study.

**Results:**

Seventy-five percent of women are delivered in a health facility with skilled providers. Women from urban areas (AOR = 1.74, *p* < 0.01), women residing in Sudurpaschim province (AOR = 5.64, *p* < 0.001), women with first parity (AOR = 2.82, *p* < 0.001), and women from rich household status (AOR = 4.60, *p* < 0.001) and women who attained at least four ANC visits (AOR = 10.81, *p* < 0.001) were associated with higher odds of delivering in a health facility. Women who were more educated and used the internet were more likely to deliver in a health facility by a skilled provider.

**Conclusion:**

Place of residence, household wealth status, and ANC visits appear to be the strongest predictors of health facility delivery with skilled providers in Nepal. Disparities in delivery in a health facility with skilled providers persist among provinces and caste/ethnicity.

**Supplementary Information:**

The online version contains supplementary material available at 10.1186/s12913-022-08822-5.

## Background

Health facility delivery is one of the essential strategies to improve maternal health and reduce the risk of maternal and child morbidity and mortality [1–4]. The increment in health facility delivery is essential for reducing maternal death from pregnancy complications [5]. Delivery in a health facility also ensures safe birth and increases the survival of mothers as well as newborn [6–8]. One of the Sustainable Development Goals (SDG) 3 is to reduce the global maternal mortality ratio to less than 70 per 100,000 live birth by 2030 [9]. Maternal mortality ratio in Nepal has decreased from 553 per 100,000 live births in 2000 to 186 per 100,000 live births in 2017, which is a great achievement, but it is still relatively high when compared to other South Asian countries excluding Afghanistan [10]. Nepal has also committed to meeting this target by 2030 [11]. In order to achieve the targets of health facility delivery, the Nepal Health Sector Strategy 2015–2020 has set a target of achieving 70% health facility delivery by 2020 [12].

Reducing maternal mortality and morbidity is a national priority in Nepal. Therefore, the government of Nepal has been implementing a safe motherhood program to reduce maternal mortality by improving maternal and child health [13]. Under the safe motherhood program, the government has included various programs such as safe abortion, family planning, ANC visits, mother safety programs, free delivery care, and transportation costs for women who are delivered in a health facility [3, 14]. Despite these efforts, Nepal has a lower percentage of women deliver in health facilities (57%) than India (87%), Maldives (95%) and Pakistan (66%) [15].

Health facility delivery is the delivery in health institutions, whether in government hospitals, public hospitals, or private hospitals/clinics. Health facility delivery enables women to receive proper medical care during childbirth and reduces the risk of maternal death. In Nepal, the percentage of women who deliver in a health facility by skilled providers gradually increased from 11% in 2001 to 58% in 2016 [16, 17] which is regarded as a significant achievement. World Health Organization (WHO) defined skilled birth attendant or skilled provider is a health professional such as doctor, nurses or midwife who has been educated and trained to provide essential care needed for women during childbirth and immediate postnatal period [18]. However, all women delivered in a health facility have not able to receive assistance from skilled providers. Among the women who were assisted by skilled provider during delivery, 97% deliver in a health facility in 2001, while this percentage has decreased to 96% in 2016 [15]. Thus, delivery in a health facility by skilled providers is a major concerned to improving maternal health.

Improvement in maternal health is the most effective way to reduce the maternal mortality ratio in the country according to the commitment to Sustainable Development Goals (SDGs). The success of the effort to reduce maternal mortality is contingent on several factors. Access to skilled providers during delivery in a health facility is essential to reducing maternal mortality. A review of literature on the subject showed that health facility delivery is influenced by demographic, socioeconomic, and contextual factors [4, 6, 7, 19–25]. The studies conducted in Nepal revealed that women's education, ANC visit [26–28], household wealth index [26–28], place of residence, women's autonomy, exposure to media [27], the distance to a health facility [28] and birth preparedness [26] were a strong predictor for health facility delivery. A recent study using the Nepal Multiple Indicator Cluster Survey 2019 focused on determinants of institutional delivery (i.e., health facility delivery) and wealth-related inequality in institutional delivery, and found that age of women, parity, four or more ANC visits, residence, sex of household head, religion, province and wealth index were the strongest determinants for health facility delivery [29]. Other studies have shown that health insurance coverage [7] and migration status [30] are also significant predictors of health facility delivery. However, these studies focused on women who delivered in a health facility for their most recent birth, including women who delivered in a health facility with a skilled provider and women who delivered in a health facility without a skilled provider, whereas health facility delivery with skilled providers is essential to achieving the SDG 3. So far, no study has explicitly focused on health facility delivery with a skilled provider. Understanding the factors that influence health facility delivery with skilled providers is critical for any intervention aimed at improving maternal health through health facility delivery. Therefore, the purpose of this study is to examine the factors influencing health facility delivery with skilled providers among women aged 15–49 years in Nepal. The findings of this study can help to design policies in Nepal to improve maternal health by promoting health facility delivery with skilled providers in Nepal.

## Methods

### Data sources

The data for this study were extracted from an individual woman and household record of the Nepal Multiple Indicator Cluster Survey (MICS) 2019 datasets. This dataset is publicly available for researchers. Permission to access and use this dataset is obtained from UNICEF/MICS website (http://mics.unicef.org/surveys). The Nepal Multiple Indicator Cluster Survey is a nationally representative cross-sectional survey carried out in 2019 by the Central Bureau of Statistics with technical and financial support from the United Nations Children's Fund (UNICEF). This survey is a part of the Global MICS program. The objective of MICS was to monitor the health status of women and children by various indicators. The survey collected information on socio-economic and demographic characteristics of households and household members, reproductive and maternal health, child mortality, child health, nutrition, literacy and education, child protection, attitudes towards domestic violence, knowledge of HIV/AIDS, access to media, and use of information and communication technology. Hence, the Nepal MICS 2019 provides internationally comparable data on a wide range of indicators about the situation of children and women. In this context, the findings of the study could help concerned authorities to promote maternal health services to achieve SDG 3.

### Sample design

The Nepal MICS employed a two-stage, stratified cluster sampling approach. In the first stage, sampling strata were identified, and then census enumeration areas (EAs) were selected from each sampling strata. In the second stage, a sample of households was selected from each selected area using a listing of households. A detailed description of the sample design is available in the Nepal MICS report [31].

### Sample size and study population

In Nepal MICS, 12,800 households were selected for the sample, of which 12,655 households were interviewed. Likewise, 15,019 women aged 15–49 years were identified as eligible for interviews. However, 14,805 women aged 15–49 years were successfully interviewed, yielding a 98.6 percent response rate. For this study, 1950 married women aged 15–49 years who had at least one live birth in the last two years preceding the survey were included in the study population.

### Study variables

The dependent variable of this study is the place of delivery for the most recent birth in the last two years before the survey. This variable is divided into two categories. Women who delivered in public and private health facilities with skilled providers are coded 1 (or 'health facility delivery with skilled providers'), and those who delivered at home or in a health facility without skilled providers are coded 0 (or 'home/elsewhere). Based on the Nepal MICS, doctor, nurse or midwife and auxiliary nurse midwife are considered as skilled providers [31].

The independent variables included in this study are: age of women, place of residence, province, women's education, parity, mother's age at most recent birth, caste/ethnicity, access to media, internet use, health insurance, migration status, household wealth quintiles, and ANC visits. The independent variables were selected based on their significance in the previous studies, empirical findings and theoretical explanations from previous studies, and variable listed in Nepal MICS report. As presented in Table 1, age of women was categorized into three groups (15–19, 20–29, and 30–49 years), women's education was broadly categorized as no education, basic (grade1-8), secondary (grade 9–12), and higher), age of mother at most recent live birth was also categorized into three groups (< 20, 20–34, and 35–49 years), caste/ethnicity was grouped into five categories— Brahman/Chhetri, other Terai caste, Dalit, Janajati and Muslim. Access to media was constructed from questions on whether a woman reads newspapers, listens to the radio, or watches television. If a woman has exposure to at least one of the three media, she is coded as 1 for exposure to media and if she has no exposure to media to any of three, she is coded as 0 for not exposed. The variable, internet use, is constructed from a question on the ever-used internet. The variable, migration status, was constructed from questions such as how long have you been continuously living in the current place and did you live in an urban or a rural area just before you move here. This variable is categorized into four groups (urban migrants, rural migrants, urban non-migrants, and rural non-migrants. The household wealth index is a composite measure of household wealth status. The household wealth index is constructed using data on dwelling characteristics, ownership of consumer goods, household assets, and housing characteristics, and ANC visits were categorized into three groups (no, 1–3 visits, and 4 + visits).

**Table 1:** Definition and categorization of study variables

Study variable	Description	Measurement
Dependent variable		
Place of delivery	Place of delivery of the most recent live birth in the two years preceding the survey	0 = Home delivery or health facility delivery without the assistance of skilled providers 1 = Health facility delivery with skilled providers/Institutional delivery with skilled providers
Independent variables		
Age of women	Age of respondents in the completed year	1 = 15–19; 2 = 20–24; 3 = 30–49
Residence	Place of residence	1 = Urban; 2 = Rural
Province	Province	1 = Province 1; 2 = Madhesh; 3 = Bagmati; 4 = Gandaki; 5 = Lumbini; 6 = Karnali; 7 = Sudurpaschim
Women's education	Highest educational level	0 = No education; 1 = Basic (Grade 1–8); 2 = Secondary (Grade 9–12); 3 = Higher
Parity	Number of births	1 = 1 birth; 2 = 2 births; 3 = 3 births; 4 = 4 or more births
Age of women at most recent live birth	Age of mother at most recent birth in the year	1 = < 20; 2 = 20–30; 3 = 35–49
Caste/ethnicity	Caste/ethnic affiliation of respondents	1 = Brahman/Chhetri; 2 = Terai caste; 3 = Dalit; 4 = Janajati; 5 = Muslim
Access to media	Exposure to media (radio, television, newspaper) at least once a week	0 = Not Exposed 1 = Exposed
Internet use	Ever used the internet	0 = No 1 = Yes
Health insurance	Health insurance for women	0 = No 1 = Yes
Migration status	Migration status of women	1 = Urban migrants; 2 = Rural migrants; 3 = Urban non-migrants; 4 = Rural non-migrants
Wealth index quintile	Household wealth index	1 = Poorest; 2 = Poor; 3 = Middle; 4 = Rich; 5 = Richest
ANC Visits	ANC visit during the pregnancy of the most recent live birth	0 = No visit; 1 = 1–3 visits; 2 = 4 + visits

### Data analysis

Data were analyzed using STATA version 15.1. Univariate analysis is carried out to analyze selected socio-demographic characteristics of women aged 15–49 years. Chi-square tests were used to check the factors that were associated with outcome variables. Bivariate logistic regression was also carried out to examine the crude association between dependent and independent variables. The backward elimination method is used to select specified variables for the logistic model based on the Bayesian Information Criterion (BIC). Based on the BIC criteria, the final logistic model included the age of women, residence, province, women's education, parity, internet use, wealth index quintile, and ANC visits. Multi-collinearity assessment is performed before the multivariate analysis. A pairwise correlation was used to examine the multicollinearity among the independent variables. After assessing multicollinearity, it was found that none of the variables were highly correlated. The results of multivariate analysis are shown in odds ratios (OR). The critical level was set at a 95% confidence interval (CI). Sample weights were applied in all analyses. The complex survey design of MICS (primary sampling units and strata) was taken in logistic regression analysis.

## Results

### Background characteristics of the respondents

Table 2 shows the background characteristics of women aged 15–49 years. The majority of the women (68%) are aged 20–29 years. About two third of women lived in an urban area while one-fifth of women were in Madhesh. About 40% of women had attained secondary while 31% received a basic education. Forty-four percent of women belonged to the first parity. About 78% of women had their recent birth at the age 20–34 years. More than one-third of women are Janajati. Sixty-two percent of women reported having access to media and 44% of women use the internet. Ninety-five percent of women have no health insurance. Seventy-one percent of women are rural migrants. More than two-thirds of women fall below the middle household wealth quintile. Seventy-eight percent of women attended at least four ANC visits for their most recent birth.

**Table 2:** Background characteristics of the respondents, Nepal MICS 2019

Characteristics	Percent	Total (N)
**Age of women**		
15–19	10.3	201
20–29	67.6	1318
30–49	22.1	431
**Residence**		
Urban	65.5	1277
Rural	34.5	673
**Province**		
Province 1	15.7	306
Madhesh	21.4	417
Bagmati	19.7	384
Gandaki	7.9	153
Lumbini	19.0	371
Karnali	6.8	132
Sudurpaschim	9.6	187
**Women's education**		
No	20.7	405
Basic	30.7	600
Secondary	39.7	775
Higher	8.8	171
**Parity**		
One	43.7	851
Two	33.0	644
Three	12.8	250
Four or above	10.5	205
**Age at most recent live birth**		
< 20	17.0	331
20–34	77.8	1517
35–49	5.3	103
**Caste/ethnicity**		
Brahman/Chhetri	27.7	540
Terai caste other	15.4	300
Dalit	15.9	310
Janajati	34.8	679
Muslim	6.3	122
**Access to media (at least once a week)**		
Not Exposed	38.1	743
Exposed	61.9	1207
**Internet use**		
No	56	1093
Yes	44	858
**Health insurance**		
No	95.2	1856
Yes	4.8	94
**Migration status**		
Urban migrants	19.5	381
Rural migrants	71	1385
Urban non-migrants	5.7	111
Rural non-migrants	3.8	73
**Wealth quintile**		
Poorest	22.7	442
Poor	21.2	414
Middle	19.7	384
Rich	19.7	384
Richest	16.7	327
**Number of ANC visits**		
No	4.5	87
1–3 visits	17.7	346
4 + visits	77.8	1517
**Facility delivery**		
Home delivery	22.5	438
Health facility delivery without skilled providers	2.2	42
Health facility delivery with skilled providers	75.4	1470
**Total**	**100.0**	**1950**

### Association between background characteristics and place of delivery

Table 3 reveals that three-fourths (75%) of women in Nepal delivered their most recent birth at a health facility with skilled providers . Delivery in a health facility with a skilled provider was higher among women aged 15–19 years (78%) and women residing in urban areas (82%). More than 80% of women of Gandaki, Bagmati, and Sudurpaschim provinces are delivered in a health facility with the assistance of a skilled provider. Similarly, the proportion of health facility delivery with a skilled provider was higher among women with secondary and higher education, who had a lower parity, whose age at most recent birth was less than 20 years, who belonged to the Brahman/Chhetri, who were exposed to media, who used the internet, who had health insurance, who were urban migrants, with highest wealth quintile, and who attended ANC at least four visits. The percentage of delivery in a health facility with skilled providers increased with the education level of women and the household wealth quintiles, and with the number of ANC visits but decreased with increasing parity.

The results of the bivariate analysis showed that place of residence, province, women's education, parity, caste/ethnicity, exposure to media, internet use, health insurance, migration status, household wealth quintile, and ANC visits were significantly associated with place of delivery at 95% confidence level (*p* < 0.001). Age of women and age at most recent birth was not found to be significantly associated with place of delivery.

**Table 3:** Percent distribution of married women aged 15–49 years by health facility delivery with skilled providers for most recent live birth in the last two years, Nepal MICS 2019

Background characteristics	%	95% CI	N	χ^2^ *p*-value
**Age of women**				
15–19	77.6	[70.2–83.5]	201	0.165
20–29	76.2	[72.8–79.3]	1318	
30–49	71.8	[67.3–75.9]	431	
**Residence**				
Urban	81.9	[78.2–85.0]	1277	0.000
Rural	63.1	[58.4–67.5]	673	
**Province**				
Province 1	77.3	[68.3–84.3]	306	0.000
Madhesh	62.0	[55.1–68.5]	417	
Bagmati	85.3	[78.1–90.4]	384	
Gandaki	85.6	[79.0–90.4]	153	
Lumbini	76.4	[69.6–82.1]	371	
Karnali	59.9	[50.0–69.2]	132	
Sudurpaschim	82.3	[75.1–87.7]	187	
**Women's education**				
No	51.6	[46.0–57.2]	405	0.000
Basic	72.6	[67.8–76.9]	600	
Secondary	85.2	[81.9–88.0]	775	
Higher	96.8	[91.8–98.8]	171	
**Parity**				
One	85.8	[82.6–88.5]	851	0.000
Two	76.8	[72.9–80.2]	644	
Three	55.8	[49.1–62.3]	250	
Four or above	51.8	[44.3–59.2]	205	
**Age at most recent live birth**				
< 20	78.9	[73.8–83.2]	331	0.059
20–34	75.1	[72.0–78.1]	1517	
35–49	67.7	[58.2–76.0]	103	
**Caste/ethnicity**				
Brahman/Chhetri	84.5	[80.4–87.9]	540	0.000
Terai caste other	62.9	[54.9–70.3]	300	
Dalit	68.6	[62.9–73.9]	310	
Janajati	78.0	[72.6–82.6]	679	
Muslim	68.0	[58.4–76.2]	122	
**Access to media**				
Not Exposed	62.8	[58.2–67.2]	743	0.000
Exposed	83.1	[80.3–85.6]	1207	
**Internet use**				
No	65.1	[61.2–68.8]	1093	0.000
Yes	88.5	[85.9–90.7]	858	
**Health insurance**				
No	74.5	[71.5–77.3]	1856	0.000
Yes	92.0	[85.7–95.6]	94	
**Migration status**				
Urban migrants	89.8	[85.6–92.9]	381	0.000
Rural migrants	71.9	[68.9–74.8]	1385	
Urban non-migrants	77.6	[64.1–87.0]	111	
Rural non-migrants	61.9	[50.2–72.4]	73	
**Wealth quintile**				
Poorest	53.8	[47.9–59.7]	442	0.000
Poor	70.9	[65.3–75.9]	414	
Middle	79.5	[74.2–83.9]	384	
Rich	86.0	[81.3–89.7]	384	
Richest	93.0	[88.4–95.9]	327	
**Number of ANC visits**				
No	15.6	[9.1–25.4]	87	0.000
1–3 visits	54.1	[48.0–60.2]	346	
4 + visits	83.7	[81.0–86.0]	1517	
**Total**	**75.4**	**[72.5–78.1]**	**1950**	

### Factors associated with health facility delivery with skilled providers

The results of the logistic regression analysis of health facility delivery with the skilled provider are presented in Table 4. Place of residence, province, women's education, parity, internet use, wealth quintiles, and ANC visits were significantly associated with health facility delivery with skilled providers. After adjusting the effects of other variables, all variables that were significantly associated in the unadjusted model remained significantly associated with health facility delivery with skilled providers. The adjusted odds ratio indicated that women living in urban areas were 1.72 times [AOR: 1.74, 95% CI: 1.24–2.43] more likely to deliver at a health facility with skilled providers than women living in rural areas. The odds of delivering in a health facility with skilled providers were 5.64 [AOR:5.64, 95% CI: 3.11–10.22] times higher among women from Sudurpaschim, 3.06 times [AOR: 3.06, 95% CI: 1.65–45.66] higher among women form Karnali, 2.32 times [AOR: 2.32, 95% 1.35–3.98] higher among women form Province 1, 2.18 times [AOR: 2.18, 95% CI: 1.25–3.80] higher among women from Gandaki and 2.07 times [AOR: 2.07, 95% CI: 1.31–3.27] higher among women from Lumbini Province compared with women from Province 2. Regarding women's education, the odds of receiving at a health facility delivery by skilled providers were 3.42 times [AOR:3.42, 95% CI:1.15–10.15] higher for women have higher education than no education. Parity was also significantly associated with health facility delivery with skilled providers in adjusted odds ratio. The odds of delivering at a health facility with skilled providers were 2.82 times [AOR:2.82, 95% CI:1.652–4.82] higher for women of one parity than women of four or more parities. Women who used the internet had significantly higher odds of delivery in a health facility by skilled provider [AOR: 1.49, 95% CI: 1.07–2.08] than women who did not use the internet. Women in the poor household status [AOR: 2.67, 95% CI: 1.69–4.22], middle household status [AOR:4.78, 95% CI: 2.89–7.90], rich household status [AOR: 5.50, 95% CI: 3.16–9.56] and richest household status [AOR: 4.60, 95% CI: 2.20–9.761] have significantly higher odds of delivery in a health facility with skilled providers than women in poorest household status. Women who attended 1–3 ANC visits and at least four ANC visits during pregnancy have 4.08 times [AOR: 1.08, 95% CI: 2.10–7.91] and 10.81 times [AOR: 10.81, 95% CI: 5.75–20.31] higher odds of delivery in a health facility with skilled providers than women who do not attend ANC visits.

**Table 4:** Logistic regression analysis of health facility delivery with skilled providers for the most recent live birth in the last 2 years preceding the survey, Nepal MICS 2019

Variables	COR	95% CI	AOR	95% CI
**Age of women**				
15–19	1.00			
20–29	0.93	0.63—1.37	1.21	0.75—1.94
30–49	0.74	0.49—1.11	1.76	0.98—3.15
**Residence**				
Rural	1.00		1.00	
Urban	2.64***	1.95—3.56	1.74**	1.24—2.43
**Province**				
Madhesh	1.00		1.00	
Province 1	2.09**	1.22—3.58	2.32**	1.35—3.98
Bagmati	3.55***	2.02—6.22	1.99*	1.16—3.40
Gandaki	3.65***	2.13—6.25	2.18**	1.25—3.80
Lumbini	1.99**	1.27—3.12	2.07**	1.31—3.27
Karnali	0.92	0.56—1.51	3.06***	1.65—5.66
Sudurpaschim	2.84***	1.70—4.76	5.64***	3.11—10.22
**Women's education**				
No education	1.00		1.00	
Basic	2.48***	1.87—3.28	1.20	0.83—1.73
Secondary	5.39***	3.94—7.36	1.46	0.98—2.15
Higher	28.75***	10.19—81.14	3.42*	1.15—10.15
**Parity**				
Four or more	1.00		1.00	
One	5.60***	3.86—8.13	2.82***	1.65—4.82
Two	3.07***	2.20—4.29	1.44	0.91—2.28
Three	1.18	0.80—1.73	0.86	0.55—1.33
**Internet use**				
No	1.00		1.00	
Yes	4.13***	3.19—5.35	1.49*	1.07—2.08
**Wealth quintile**				
Poorest	1.00		1.00	
Poor	2.09***	1.51—2.88	2.67***	1.69—4.22
Middle	3.32***	2.26—4.86	4.78***	2.89—7.90
Rich	5.27***	3.45—8.05	5.50***	3.16—9.56
Richest	11.40***	6.24—20.82	4.60***	2.20—9.61
**ANC visits**				
No	1.00		1.00	
1–3 visits	6.37***	3.36—12.06	4.08***	2.10—7.91
4 + visits	27.62***	14.80—51.57	10.81***	5.75—20.31

*COR* Crude odds ratio (unadjusted), *AOR* Adjusted odds ratio

^***^ *p* < 0.001, ** *p* < 0.01, * *p* < 0.05

## Discussion

The Government of Nepal has implemented various strategies to improve maternal and newborn health. This study explored the factors associated with delivery in a health facility with skilled providers among women aged 15–49 years in Nepal. The results of this study showed that 75% of women delivered in a health facility. This result was, however, higher than that of Bangladesh [32]. Previous studies indicate that 59% of women are delivered in a health facility in Nepal [26]. This finding suggests that the increasing proportion of women who delivered in a health facility could be attributed to the various efforts and interventions that have been implemented by the Government of Nepal to improve delivery in a health facility. These efforts include expanding the birthing center, cash incentives, free delivery services, and incentives for 4 ANC visits [33]. In addition, Free Delivery Care (FDC) policies have a more significant impact on improving access to and use of health facilities for delivery [34]. This study found that place of residence, province, parity, caste/ethnicity, household wealth status, and ANC visits significantly influenced delivery in a health facility with skilled providers.

The study found that women's delivery in a health facility with a skilled provider is influenced by their place of residence. Women in an urban setting are more likely to deliver in a health facility. This finding is in line with previous studies from Nepal [35], India [25, 36], Indonesia [24], Ethiopia [20], Kenya [37], and Ghana [7, 38]. This finding could be attributed to easier access to maternal health services in urban areas than in rural areas in Nepal. There are great differences in access to maternal health services between urban and rural areas. A previous study in Nepal [39] and South Asia [40] suggested that women belonging to poor families, fewer health facilities, and lack of reliable transportation to the health facility in rural areas may be contributing to less utilization of health facility delivery. However, another study in Nepal did not find a significant association between delivery in a health facility and place of residence after adjusting the covariates [26].

This study revealed that provincial variation is an important predictor of delivery in a health facility with skilled providers. Following previous studies in Nepal [26, 28, 35], this study found that there has been a significant provincial difference in delivery in a health facility with skilled providers. This study indicated a significant difference between provinces in the use of health facilities for delivery. Madhesh Province was found to be disadvantaged, and Sudurpaschip was found to be better off in terms of delivery in a health facility with skilled providers followed by Karnali, Province 1, and Lumbini Province. The unequal utilization of health facilities for delivery across provinces may be associated with disparities in the availability and utilization of maternal health services across the provinces [41]. While the progress made in reducing inequality in utilization of maternal health services, expansion of birthing centers and emergency obstetric centers, and better socioeconomic development in Sudurpaschip may be attributed to a greater likelihood of delivery at a health facility [42]. However, there are limited studies that show the relationship between province and health facility delivery.

In line with previous studies [27, 28, 43], women's education have a significant association with delivery in a health facility by skilled providers. The possible explanation for this result is that educated women may have greater capcity to decision making regarding delivery in health facility by skilled providers. The study reveals that women who had only first birth (first parity) were more likely to deliver in a health facility with skilled providers. This finding is consistent with previous studies in Nepal [44, 45], Ethiopia [46, 47], Ghana [7, 38, 48], and Senegal [49]. The possible explanation could be that women with higher parity may have the self-confidence to deliver at home and not prefer to deliver in a health facility. However, first parity women may be afraid of complications in pregnancy and childbirth. Therefore, first parity women were more likely to deliver in a health facility with skilled providers.

Similar to the previous studies in Senegal [49], Kenya [50], Malawi [51], and India [52], this study identified that women with high household wealth were more likely to give birth in a health facility with skilled providers compared with those in poor household wealth. Previous studies in Nepal [26, 28, 30, 35, 45] and low- and middle-income countries [53] found that the household wealth index was positively associated with delivery in a health facility. Women who belong to higher household status have been educated and aware of the importance of health facility delivery and have the financial capacity to pay travel and medical care expenses if necessary [54, 55]. The strong association of household wealth status with health facility delivery with skilled providers suggests that poor economic status is a barrier to health facility delivery. To reduce this barrier, the Government of Nepal has promoted safe motherhood through incentives such as free delivery care and transportation incentives scheme for women of poor households for delivering in a health facility.

This study found that the number of ANC visits was significantly associated with delivery in a health facility with skilled providers. Women who attended at least one ANC visit were more likely to deliver in a health facility with skilled providers. This result is consistent with previous studies conducted in Ethiopia [20, 56], Ghana [20], Nigeria [57], and Mozambique [43]. Previous studies in Nepal [3, 39, 45, 58, 59] demonstrated that ANC visits as a strong predictor of delivery in a health facility. Women who attended more ANC visits were more aware and had more information about danger signs during pregnancy. ANC visits allow mothers to contact health personnel frequently and develop positive attitudes towards delivery in a health facility.

The main strength of this study is that it used the Multiple Indicator Cluster Survey, which is a major source of data on over 30 Sustainable Development Goals indicators and more than 176 MICS indicators [31]. and allows for the generalization of the finding at the national level. This study did not include some potential factors that may affect the delivery in a health facility such as religion, employment status of women, and distance to the health facility, which could be the further area of study. The purposively selected independent variables are included in the study. The findings of the study can be generalized to the county.

The Nepal Multiple Indicator Cluster Survey provides cross-sectional data. This type of data can be used to describe the prevalent characteristics and relationship between selected independent variables and outcome variables but not to analyze cause-and-effect relationships between different variables.

## Conclusion

The study reveals that the prevalence of delivery in a health facility with skilled providers is low among the women who reside in a rural area, who live in Karnali Province, who are less educated, who have four or more parity, who have not used the internet, who belong to poor household wealth status and who do not attend ANC visits. To improve delivery in a health facility, emphasis must be given to 4 + ANC visits among women who reside in rural areas and belong to poor households. Hence, the study recommends concerned authorities to expand and promote maternal health services to women from poor household status, especially those who are residing in rural areas–considering the findings of this research. In addition to this, efforts to reduce provincial inequalities in maternal healthcare services are needed to improve delivery in a health facility with skilled providers.

## Supplementary Information

**Additional file 1.**

## Data Availability

The data used in this study are freely accessible at the MICS website http://mics.unicef.org/surveys. We agree to the attached terms and conditions for the data-sharing policy.

## Abbreviations

ANC: Antenatal care
AOR: Adjusted odds ratio
CI: Confidence interval
DHS: Demographic and Health Survey
EA: Enumeration
MICS: Multiple Indicator Cluster Survey
NDHS: Nepal Demographic and Health Survey
OR: Odds ratio
SBA: Skilled birth attendant
SDG: Sustainable development goal
SLC: School living certificate
UNICEF: United Nations Children's Fund
